# Effect of corneal stromal pocket irrigation in small-incision lenticule extraction

**DOI:** 10.1038/s41433-020-0840-1

**Published:** 2020-03-10

**Authors:** Han Wang, Hui Ding, Bo-wen Ouyang, Zhenduo Yang, Tan Zhong, Hongming Fan, Xingwu Zhong

**Affiliations:** 1 State Key Laboratory of Ophthalmology, Zhongshan Ophthalmic Center, Sun Yat-sen University, Guangzhou, 510060 China; 2 Hainan Eye Hospital, Zhongshan Ophthalmic Center, Sun Yat-sen University, Haikou, 571152 Hainan Province China

**Keywords:** Refractive errors, Outcomes research, Surgery

## Abstract

**Objectives:**

To investigate the effect of corneal stromal pocket irrigation after small-incision lenticule extraction (SMILE) on visual acuity, intraocular pressure (IOP), corneal parameters and complications after surgery.

**Methods:**

A total of 242 eyes of 121 patients undergoing SMILE were enrolled in this prospective controlled study, and it was designed for one eye to randomly undergo SMILE with balanced salt solution irrigation of the corneal stromal pocket, while the other eye was not. The uncorrected distance visual acuity (UDVA) and slit lamp examination were recorded at 1 hour, 1 day, 1 week, and 1 month. Postoperative corneal density, corneal biomechanical, corneal endothelial cell number, and anterior OCT images were compared at 1 day, 1 week, and 1 month.

**Results:**

Compared with the nonirrigation group, the irrigation group showed significantly higher UDVA at 1 day postoperatively (*P* < 0.05), but there was no significant difference during the rest of the postoperative period (1 hour, 1 week, and 1 month). In addition, no significant differences were found in IOP, corneal density, corneal biomechanics, corneal endothelial cells, and corneal morphology. No visual decline or severe postoperative complications were found in the patients in this study.

**Conclusions:**

Interlamellar irrigation did not affect IOP, corneal parameters, morphology, complications, or UDVA at 1 hour, 1 week, and 1 month after the operation, but it may promote UDVA 1 day after the operation.

## Introduction

As a popular area of research in the field of corneal refraction, the safety, effectiveness, and predictability of small-incision lenticule extraction (SMILE) surgery have been confirmed by a large number of studies [1] with high patient satisfaction [2]. However, SMILE also has higher demands for the operator and a steeper learning curve than does traditional LASIK surgery [3]. It is also difficult to perform due to the optical lens being too thin for low myopia [4]. Moreover, due to the limitations of femtosecond lasers, the lens may still have a tissue bridge connection with adjacent tissues, resulting in partial lens separation difficulty, intraoperative epithelial damage, incision tears, and other complications [5]. Clinical findings and some prospective studies showed that among a subset of early postoperative patients with water mist, subjective vision volatility and delayed early vision restoration were more common after SMILE than after LASIK and FS-LASIK [6, 7]. Nonetheless, the specific reason for these differences has not been fully elucidated. Current reports suggest that these issues may be related to the decrease in light scattering and front elastic layer microwrinkles caused by femtosecond laser cutting in the corneal stroma [8].

Unlike LASIK and FS-LASIK, SMILE does not incorporate intracapsular flushing of the corneal stroma after the removal of microlenses into the standard operation procedure. Most surgeons believe that irrigation can limit corneal epithelium growth into the cornea along the edge of the corneal incision, remove inflammatory factors and reduce infectious microorganisms during surgical procedures; thus, it can reduce the risk of infection after surgery [9–12]. We suspect that the pocket interface after irrigation is smoother and that adherence to the upper and lower contact surfaces is better, thus contributing to early visual recovery. Nevertheless, some researchers believe that in SMILE, the lateral incision is small, which may cause the irrigation fluid to accumulate in the corneal stroma pocket and not easily drain out, causing early postoperative corneal stromal oedema and delay visual recovery [13]. Moreover, the possibility of infection and foreign body retention after irrigation may be increased due to contamination of fluids and excessive manipulation.

In SMILE, it is unclear whether irrigating the stromal pouch will increase the risk of infection after operation and affect early vision and other corneal-related parameters. At present, there is no relevant report regarding this controversy. Therefore, we conducted a randomized, prospective, double-blind, controlled study on intraoperative corneal stroma pocket irrigation in SMILE. In this study, we aimed to demonstrate the effects of corneal stromal pouch irrigation on corneal density, morphology, biomechanics, and endothelium. In addition, we sought to evaluate early visual acuity and infection risk after SMILE.

## Subjects and methods

This prospective controlled study was completed at the Hainan Eye Hospital of the Zhongshan Ophthalmic Center in China from February to November 2018. Two hundred forty-two eyes of 121 patients undergoing SMILE for the treatment of myopia were recruited in the hospital setting. The study adhered to the tenets of the Declaration of Helsinki and was performed in accordance with good clinical practices and local regulatory requirements. The protocol was reviewed and approved by the Ethics Review Board of Hainan Eye Hospital of the Zhongshan Ophthalmic Center. All patients signed the consent form before surgery. This study was registered in the Chinese Clinical Trial Registry (No. ChiCTR1900022049).

Subjects needed to meet the following criteria: stable myopic refractive status for more than 2 years, 18 years of age or older, binocular central corneal thickness ranging from 490 to 640 μm, and manifest spherical equivalent between −1.50 and −10.00 D, with manifest cylinders <3.0 D. All subjects were free of complicated systemic diseases (such as diabetes, hypertension, heart disease, hyperthyroidism, and scarring) or a history of other ocular pathology. Before the examination, patients needed to stop using a corneal contact lens (soft contact lenses ≥2 weeks, RG ≥1 month, and orthokeratology ≥3 months). Patients with other ocular diagnoses, including amblyopia, keratoconus, glaucoma, central corneal residual stromal bed thickness <260 μm, or active ocular surface inflammation, were excluded from the study.

### Randomization

The randomization was performed by a random number table. A total of 242 eyes from 121 patients underwent an SMILE procedure and were randomly divided into the following groups: SMILE with irrigation and SMILE without irrigation. The randomization protocol was implemented by an informed refractive surgeon when the corneal stroma lenticule was separated. Only the left eye was irrigated with balanced salt solution (BSS) in 61 patients during SMILE surgery, while only the right eye was irrigated in the remaining 60 patients. One eye in each patient served as a control to minimize clinical, lifestyle habits, or environmental factors.

### Surgical techniques

All patients received routine preoperative topical antibiotic eye drops (0.5% levofloxacin eye drops (Cravit; Santen Pharmaceuticals, Japan)) four times a day for 3 days. Topical anaesthesia with proparacaine hydrochloride eye drops (Alcaine; Alcon Laboratories, USA) was administered before surgery. Surgery on both eyes of all patients was completed by the same experienced surgeon using the Visumax femtosecond laser system (Carl Zeiss, Germany) at a power setting of 500 kHz with SMILE system software. The parameters of cap thickness were 110–120 mm (all patients had the same cap thickness in both eyes), and the lenticule diameter in the two groups was 6.5 mm. The laser cut energy was 140 nJ, and the side cut for accessing the lenticule was 90° at a circumferential width of 2.0 mm. After the femtosecond laser treatment was completed in both eyes of the patient, the corneal stroma lenticule was separated under the microscope of the surgical operating table. The corneal pocket and ocular surface around the corneal incision were irrigated with 1.0 mL BSS using a 10 mL syringe for SMILE in the irrigation group, while the SMILE without irrigation group received irrigation of the ocular surface alone, and corneal pocket irrigation was not performed. All patients wore bandage contact lenses after surgery. Routine anti-inflammatory treatment was performed after the surgery, and levofloxacin eye drops were prescribed four times daily for 1 week; 0.3% tobramycin combined with 0.1% dexamethasone eye drops (Tobradex; Alcon Laboratories, USA) were prescribed four times daily for 3 days; 0.5% loteprednol etabonate ophthalmic suspension (Lotemax; Bausch & Lomb Incorporated, USA) was prescribed four times daily for 4–14 days; and 0.1% sodium hyaluronate eye drops (Hialid; Santen Pharmaceuticals, Japan) were prescribed four times daily for 1 month.

### Clinical examinations

All patients had postoperative study assessments including slit lamp examinations and uncorrected distance visual acuity (UDVA) evaluations at 1 h, 1 day, 1 week, and 1 month after surgery. The corrected intraocular pressure (IOP) and corneal biomechanics measured by Corvis ST (Oculus, Germany), corneal densitometry measured by Pentacam HR (Oculus, Germany), corneal endothelial cells measured by SP-3000p (Topcon, Japan), and anterior segment optical coherence tomography (AS-OCT, Carl Zeiss, Germany) were evaluated at 1 day, 1 week, and 1 month after surgery.

### Statistical analysis

Data are expressed as the mean ± standard deviation (SD) or as median ± interquartile range (Q2 ± IQR). Significant differences in parameters between the two groups were calculated using paired *t*-tests or the Wilcoxon signed-rank test. Values <0.05 were considered statistically significant, and statistical calculations were performed using SPSS Software 19.0 (SPSS, Inc., Chicago, IL).

## Results

### Demographic data

Two hundred forty-two eyes from 121 patients (76 males and 45 females) were recruited for the study. The average age was 23.4 years (range 18–34 years). Preoperative parameters are given in Table 1. There were no significant differences in terms of these parameters (all *P* > 0.05).

**Table 1:** Demographic characteristics of patients in both groups preoperatively (mean ± SD or Q2 ± IQR).

Parameter	Mean ± SD or Q2 ± IQR		*P* value
	SMILE with irrigation (*n* = 121)	SMILE without irrigation (*n* = 121)	
Pre-BCDVA (LogMAR)	−0.01 ± 0.04	−0.02 ± 0.03	0.39
Pre-sph (D)	−4.89 ± 1.85	−4.93 ± 2.15	0.87
Pre-cyl (D)	−0.75 ± 0.75	−0.75 ± 0.50	0.65
CCT (µm)	548.46 ± 31.70	549.97 ± 32.26	0.71
IOP (mmHg)	15.50 ± 3.25	16.00 ± 3.50	0.80

*logMAR* logarithm of the minimum angle of resolution, *BCDVA* best corrected distance visual acuity, *CCT* central corneal thickness.

### Efficacy

All patients were followed up within 1 month after the operation. Table 2 shows the time course of the visual parameters after SMILE between the two groups. There were no significant differences in postoperative logMAR UDVA between the groups at 1 h, 1 week, and 1 month after surgery (*P* = 0.26, *P* = 0.54, and *P* = 0.63, respectively). However, the postoperative logMAR UDVA of the SMILE with irrigation group was significantly better than that of the SMILE without irrigation group 1 day after surgery (*P* = 0.02). Within 1 h after surgery, the number of eyes with better eyesight among irrigated eyes was 46, and the number among unirrigated eyes was 38, while other eyes exhibited consistent vision. Among the patients, visual acuity fluctuated greatly (the difference in visual acuity was >4 lines in the eye chart); four patients had better eyesight in the irrigated eye, and three patients had better eyesight in unirrigated eye. One day after surgery, 38 irrigated eyes had better visual acuity recovery than unirrigated eyes, while 26 had better UDVA recovery without irrigation. Among the patients with large differences in vision, six patients with irrigated eyes had better visual acuity than did those with unirrigated eyes. In contrast, only one patient had better eyesight without irrigation.

**Table 2:** Comparison of visual parameters in the postoperative period (1 h, 1 day, 1 week, and 1 month) (mean ± SD).

Timepoint	Mean UDVA (logMAR) ± SD		*P* value
	SMILE with irrigation (*n* = 121)	SMILE without irrigation (*n* = 121)	
1 h	0.18 ± 0.16	0.21 ± 0.18	0.26
1 day	−0.05 ± 0.09	−0.02 ± 0.11	**0.02**
1 week	−0.09 ± 0.07	−0.08 ± 0.06	0.54
1 month	−0.10 ± 0.06	−0.10 ± 0.07	0.63

*UDVA* uncorrected distance visual acuity.

### Safety

During follow-up, no serious complications occurred, such as cap rugs, epithelial cell implantation inside the incision, or transient photosensitivity syndrome. Only two eyes had DLK grade I 1 day after the operation, with one in the irrigation group and another in the nonirrigation group. However, both patients improved by 1 week after the operation. The opaque bubble layer was observed in 26 of 121 eyes (21%) in the SMILE with irrigation group, while 24 were observed in the SMILE without irrigation group (*P* > 0.05). However, the difference was nonsignificant.

### Corneal densitometry

As previously described [14], corneal density measurements are divided into four regions (0–2, 2–6, 6–10, and 10–12 mm) according to the diameter, and each area is divided into the following layers: anterior (the most anterior 120 μm of the cornea), central, and posterior (the most posterior 60 μm of the cornea). The optical area of the operation is <6.5 mm, and the parameters of the 6–10 mm area are greatly influenced by factors such as eyelash and eyelid occlusion. Therefore, we mainly compared the corneal density differences between different layers of 0–2 and 2–6 mm in these two different regions. The density values at different levels in different regions of the two groups (irrigation or nonirrigation) were measured in grayscale units and are summarized in Table 3. Regarding values preoperatively and 1 day, 1 week, and 1 month postoperatively, no significant difference was found between the two groups. Moreover, there was no significant difference between the two groups in the anterior, middle, and posterior corneal levels (all *P* > 0.05).

**Table 3:** Corneal densitometry in different corneal layers in both groups (presented in grayscale units, GSU).

Variable	Mean ± SD		*P* value
	SMILE with irrigation (*n* = 121)	SMILE without irrigation (*n* = 121)	
Preoperative			
Anterior 0–2 mm	23.34 ± 1.53	23.37 ± 1.61	0.91
Central 0–2 mm	13.25 ± 0.87	−13.26 ± 0.93	0.91
Posterior 0–2 mm	11.35 ± 0.85	11.27 ± 0.87	0.50
0–2 mm total	15.99 ± 0.93	15.97 ± 1.01	0.87
Anterior 2–6 mm	20.71 ± 1.40	20.70 ± 1.41	0.95
Central 2–6 mm	11.74 ± 0.68	11.75 ± 0.71	0.97
Posterior 2–6 mm	10.22 ± 0.66	10.14 ± 0.71	0.40
2–6 mm total	14.24 ± 0.83	14.20 ± 0.83	0.70
1 day			
Anterior 0–2 mm	27.55 ± 2.15	28.09 ± 2.69	0.08
Central 0–2 mm	14.26 ± 1.10	14.34 ± 1.14	0.56
Posterior 0–2 mm	11.06 ± 0.85	11.03 ± 0.91	0.80
0–2 mm total	17.63 ± 1.22	17.82 ± 1.40	0.25
Anterior 2–6 mm	24.49 ± 2.14	25.05 ± 2.38	0.06
Central 2–6 mm	12.33 ± 0.81	12.43 ± 0.84	0.35
Posterior 2–6 mm	10.16 ± 0.68	10.18 ± 0.70	0.79
2–6 mm total	15.66 ± 1.11	15.91 ± 1.18	0.09
1 week			
Anterior 0–2 mm	26.84 ± 2.15	26.90 ± 2.39	0.84
Central 0–2 mm	14.13 ± 1.00	14.05 ± 1.00	0.57
Posterior 0–2 mm	11.15 ± 0.84	11.16 ± 0.94	0.95
0–2 mm total	17.37 ± 1.18	17.36 ± 1.28	0.94
Anterior 2–6 mm	23.80 ± 2.38	23.99 ± 2.25	0.56
Central 2–6 mm	12.36 ± 1.18	12.24 ± 0.74	0.33
Posterior 2–6 mm	10.27 ± 0.68	10.28 ± 0.72	0.94
2–6 mm total	15.48 ± 1.08	15.50 ± 1.20	0.88
1 month			
Anterior 0–2 mm	24.07 ± 3.35	24.16 ± 3.39	0.73
Central 0–2 mm	13.82 ± 0.95	13.83 ± 1.02	0.98
Posterior 0–2 mm	11.24 ± 0.91	11.38 ± 1.41	0.41
0–2 mm total	16.37 ± 1.63	16.42 ± 1.17	0.77
Anterior 2–6 mm	21.47 ± 1.59	21.51 ± 1.58	0.85
Central 2–6 mm	12.05 ± 0.63	11.93 ± 1.36	0.42
Posterior 2–6 mm	10.36 ± 0.64	10.38 ± 0.66	0.82
2–6 mm total	14.62 ± 0.87	14.65 ± 0.88	0.84

### Corneal biomechanics and corneal thickness

Corneal biomechanics were measured with a Scheimpflug imaging technology system (Corvis ST: software version 1.2r1126); we and other researchers have previously described its working principle and application in detail [15, 16]. In the SMILE with irrigation group, the IOP values measured by Corvis ST were 13.84 ± 2.31, 13.36 ± 2.13, 13.45 ± 2.42, and 11.39 ± 1.78 preoperatively, 1 day, 1 week, and 1 month postoperatively, respectively, whereas the IOP values were 13.90 ± 2.35, 13.21 ± 2.13, 13.41 ± 2.80, and 11.48 ± 1.80 in the SMILE without irrigation group (*P* = 0.77, *P* = 0.53, *P* = 0.93, and *P* = 0.83, respectively). Based on the corneal thickness measured by Corvis ST in both groups at different time points for inspection, the trends were 554.03 ± 30.47, 464.71 ± 39.84, 458.91 ± 40.26, and 457.65 ± 38.61 preoperatively, 1 day, 1 week and 1 month postoperatively, respectively, in the irrigation group; those in the SMILE without irrigation group were 554.75 ± 30.51, 464.73 ± 40.12, 457.28 ± 40.24, and 457.24 ± 38.60 (*P* = 0.85, *P* = 0.78, *P* = 0.81, and *P* = 0.90, respectively). There was no significant difference in corneal thickness between the two groups at different time points.

**Fig. 1:**
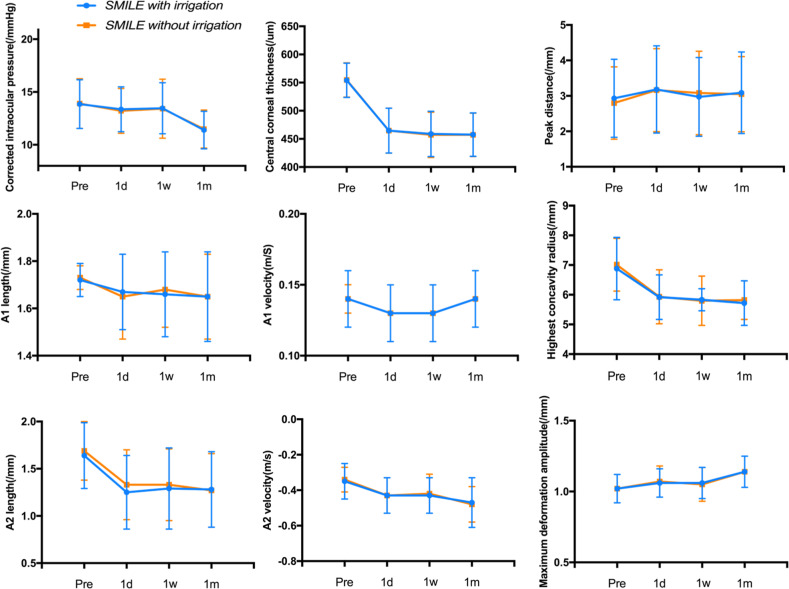
Comparisons of crude measurements of corneal biomechanical parameters between the irrigation and nonirrigation groups (all *P* > 0.05). Peak distance = distance between both nondeformed peaks; A1 velocity = velocity at the first applanation; A1 length = length at the first applanation; highest concavity radius = central radius of curvature at the highest concavity; A2 length = length at the second applanation; A2 velocity = velocity at the second applanation; maximum deformation amplitude = maximum deformation amplitude of the apex.

We also compared corneal biomechanical parameters, including A1L, A1V, A2L, A2V, PD, HCR, and MDA, between the two groups at the following different time points: before surgery and 1 day, 1 week, and 1 month after surgery. The results showed a high degree of consistency between the two different treatment groups, with no significant difference (all *P* > 0.05) (Fig. 1).

### Corneal endothelial cells

SP-3000P, a noncontact measurement system for corneal endothelium imaging, was used to observe and record the morphological structure and number of corneal endothelial cells. We used manual image acquisition mode to compare corneal density at four different time points in the same part for each acquisition. In the irrigation group, corneal density was 2874.6 ± 212.6, 2893.0 ± 232.6, 2948.8 ± 252.7, and 2923.5 ± 184.3. In the nonirrigation group, corneal density was 2881.7 ± 236.2, 2908.9 ± 200.3, 2866.4 ± 198.2, and 2833.5 ± 268.4. No statistically significant difference was found in any of the endothelial cell indexes (*P* = 0.98, *P* = 0.63, *P* = 0.09, and *P* = 0.21, respectively; all *P* > 0.05).

### Cross-sectional morphology of the cornea evaluated by AS-OCT

**Fig. 2:**
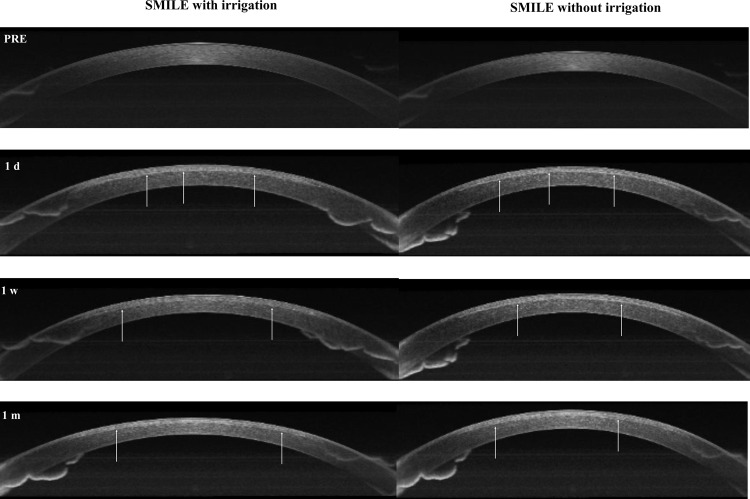
Comparisons of corneal cross section morphology between two groups. Preoperative and postoperative (3 time points) examination of AS-OCT for one patient who underwent SMILE (white arrowhead shows the interstromal space after lenticules removal).

AS-OCT examination provided images of the cornea of the eyes at three examinations within 1 month after operation (Fig. 2). The patient’s eyes were clearly visible in both the irrigated and nonirrigated groups, and the hyperreflective lines of the interlaminar space and surgical incision margin were observed. Over time postoperatively, the reflectivity gradually decreased; however, the difference in reflectivity among the groups was nonsignificant.

## Discussion

As one of the mainstream corneal refractive surgeries, SMILE has no flap and is minimally invasive, which better protects the integrity of the corneal nerve and reduces the biomechanical influence on the cornea. SMILE avoids the intraoperative and postoperative complications of the corneal flap. In addition, SMILE reduces the incidence and duration of postoperative dry eye syndrome [3, 5], and many studies have demonstrated this result by measuring corneal sensation and corneal nerves under confocal microscopy [6, 7]. At present, there is no uniform standard for intracapsular flushing in SMILE surgery, and the effect of intracapsular flushing on early visual quality and corneal biomechanics has rarely been reported.

In this study, we performed SMILE on randomized eyes with and without irrigation in the corneal stroma pocket to assess the influence of different operations on visual function and corneal biomechanics at the very early stage (1–24 h postoperatively) and the stage of gradual stabilization (1 week to 1 month). UDVA did not differ between the two groups at 1 h, 1 week, and 1 month after surgery. One day after the operation, the visual acuity of the irrigation group was significantly better than that of the nonirrigation group. In Alex et al. study [12], they also believed that 2 ml BSS flushing could achieve good results. The flushing fluid may reduce the wrinkles in Bowman’s layer and make the two layers of the stromal pocket more adherent to each other. One day after the operation, both the irrigated group and nonirrigated group achieved better UDVA (more than +0.10[LogMAR]). Although flushing eyes can result in better vision, the improvement in visual acuity is not obvious (no more than three rows of the visual chart). In addition, the effect is not stable. Individual patients had better visual acuity in the nonirrigated eyes. Considering that some patients are currently undergoing SMILE surgery for physical examination, flushing may not be a safe method for rapid visual recovery. To determine whether the irrigation solution would accumulate in the corneal layer, causing corneal oedema and even affecting the corneal endothelium and biomechanical properties, we compared the corneal density of different areas at four different time points before and after surgery in our study. The density of the anterior/central cornea increased significantly, and the density of the posterior cornea did not change much at 1 day after surgery compared with that before surgery in the 0–2 mm or 2–6 mm areas. At 1 week, corneal density gradually decreased, and it tended to be normal at 1 month. However, in all different areas and levels, there was no significant difference between the irrigated group and nonirrigated group. Corvis ST also demonstrated no significant difference in corneal thickness between the two groups at different time points. This finding may suggest that corneal oedema and increased thickness caused by water infiltration can be resolved in a short time without affecting vision and refraction in the future. We can also speculate that the early oedema after surgery may be due to lenticule extraction rather than the accumulation and absorption of flushing fluids. This idea is also consistent with the basic research results of Liu et al. [13]. Compared with the preoperative images, the postoperative corneal cross-sectional images provided by AS-OCT also suggested that the density and reflectivity of the stroma increased significantly, and irrigation did not cause corneal stromal oedema or morphological changes. Interestingly, in our usual clinical work, we observed that some patients retained a sensation of water fog 1 week after surgery, whereas almost all patients had no such feeling after 1 month; our findings may explain this sensation.

At different time points before and after surgery, the density of the posterior layer of the cornea did not change significantly. In addition, we observed no significant difference in the corneal endothelium. This result may show that irrigation does not cause swelling or decrease the number of corneal endothelial cells. Changes in corneal thickness can lead to changes in corneal deformation amplitude [17]. To investigate whether flushing affects corneal deformation amplitude, we used Corvis ST to dynamically monitor various parameters of corneal biomechanics, and there was no significant difference between the two groups.

One relevant question is whether irrigation increases the possibility of foreign body retention or reduces the production of inflammatory factors. None of our subjects had serious complications (epithelial cell implantation inside the incision, severe dry eye or transient photosensitivity syndrome). There was a mild degree of DLK in both the irrigated group and nonirrigated group, and all patients improved within a week. Therefore, we suggest that irrigation might not be associated with postoperative complications. However, given the prospective clinical research of this study, it was not possible to increase the amount of data recorded postoperatively. However, a more complete study (with contrast sensitivity, aberration, and MTF cut off) and a larger sample size are needed. To investigate whether flushing can reduce the risk of infection, this hypothesis should be examined in further studies. Moreover, whether different flushing fluids with different properties, such as hormones or antibiotics, at different doses can produce different results need further study.

In conclusion, in this prospective, double-blind, controlled experiment, compared with no irrigation, irrigation of the corneal stroma pocket with 1 ml BSS in one eye resulted in no significant differences in corneal morphology/density/biomechanics or postoperative complications. There was no significant change in visual acuity at 1 h, 1 week, and 1 month after the operation and the visual acuity of the irrigation group was better than that of the nonirrigation group at 1 day after the operation.

## Summary

### What was known before

- Our prospective, double-blind, controlled experiment, compared with no irrigation, irrigation of the corneal stroma pocket with 1 ml BSS in one eye resulted in no significant differences in corneal morphology/density/biomechanics or postoperative complications.
- There was no significant change in visual acuity at 1 h, 1 week, and 1 month after the operation and the visual acuity of the irrigation group was better than that of the nonirrigation group at 1 day after the operation.

### What this study adds

- Provide experimental basis for whether SMILE is performed during irrigation and its postoperative performance.
